# Enlarged Epitrochlear Glands

**Published:** 1915-02

**Authors:** 


**Enlarged Epitrochlear Glands.** Editorial, Paediatrics, Oct. 1914. In over 1200 consecutive dispensary cases of all ages and both sexes, both glands were found enlarged in 65%; one in 10% more. Various local and general infections, leucocythaemia, pseudo-leucocythaemia, etc., accounted for some of the cases, in others no cause could be determined. Syphilis certainly did not account for more than 10% of all. Hence the common teaching that this sign is diagnostic of syphilis is incorrect.

